# HPTLC-Densitometric Determination of Cetirizine and Montelukast Analysis in Combined Tablet Dosage Forms

**Published:** 2013

**Authors:** Saeed Haghighi, Mahmoud Reza Shapouri, Mitra Amoli-Diva, Kamyar Pourghazi, Hossein Afruzi

**Affiliations:** a *Quality Control laboratory, Darou Pakhsh Mfg. Co., Tehran, Iran.*; b *Faculty of Chemistry, Kharazmi (Tarbiat Moalem) University, Tehran, Iran.*; c *Inistitue for Higher Education, Parseh, Tehran, Iran. *; d *Faculty of Sciences, Lorestan University, Khoramabad, Iran. *

**Keywords:** Cetirizine, Montelukast, HPTLC, Quantitative Analysis, Densitometry

## Abstract

A simple, accurate and rapid high performance thin layer chromatography (HPTLC)- densitometric method was developed for separation and determination of cetirizine (CET) as a long acting antihistamine and montelukast (MON) as an antileukotriene in pharmaceutical dosage forms. The compounds were separated on silica gel 60 F_254_ HPTLC plates using a mixture of ethyl acetate : methanol : ammonia solution (25%) (14 : 3 : 2 v/v/v) as mobile phase. The plates were developed vertically up to a distance of 80 mm. Compact spots of both cetirizine (R_f_ = 0.30 ± 0.01) and montelukast (R_f_ = 0.52 ± 0.02) were obtained. UV detection was performed at 230 nm. Quantitative analysis was performed by absorbance densitometry using peak area. The method was validated in terms of linearity, precision, accuracy, limit of detection (LOD), and limit of quantification (LOQ). The calibration curves were linear in the range of 40-2000 ng spot^-1^ for cetirizine and 120-1000 ng spot^-1^ for montelukast. For MON, recovery varied in range of 99.20-100.88% with RSD ranging from 1.02 to 1.90% and for CET, recovery varied in range of 98.13-100.05% with RSD ranging from 1.57 to 1.85%. The LODs were found to be 3.94 and 2.08 ng spot^-1^ for CET and MON, respectively. It was observed that the proposed HPTLC method could be used for efficient analysis and monitoring of the CET and MON in combined tablet dosage forms, more convenient with better precision and accuracy than HPLC method.

## Introduction

**Figure 1:**
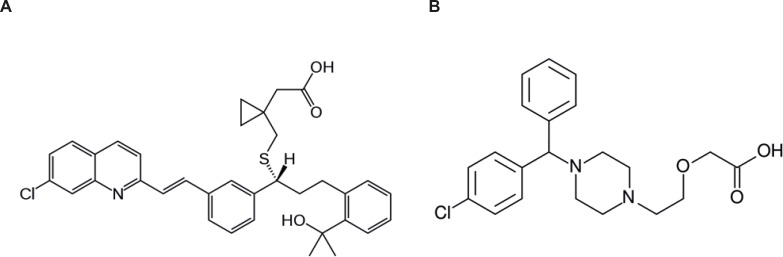
Chemical structures of montelukast (A) and cetirizine (B).

Leukotriene inhibitors are a new pharmacological class of compounds for asthma management. Montelukast (MON) (Figure 1a) is a potent leukotriene receptor antagonist known as [R-(E)]-1-[[[1-[3-[2-(7- Chloro-2-quinolyl)] ethenyl] phenyl]-3-[2-(1- hydroxy-1-methyl-ethyl) phenyl]-propyl] thio] methyl] cyclopropaneacetic acid, (trade name Singulair) used for treatment of seasonal allergic rhinitis and asthma (1). Its empirical formula is C_35_H_36_ClNO_3_S and usually administered orally. MON is the only leukotriene modifier approved by US Food and Drug Administration for being used by children from 2 to 12 years old (2). 

Various analytical methods have been reported for the assay of MON in the dosage forms or in plasma. Although most of them rely on the use of chromatographic methods such as HPLC(3-5), HPLC with fluorescence detection (6-10), stereoselective HPLC (11), and HPTLC (12), other methods including capillary electrophoresis and voltammetric determination were also used (13, 14).

Cetirizine (CET), Figure 1b, is a long acting antihistamine with some mast-cell stabilizing activity which is known as 1-(2-(carboxymethyl) ethyl)-4-(4-chlorobenzhydryl) piperazinium dichloride (trade name Zyrtec) and widely used in the comprehensive management of allergic rhinitis, the symptoms of which include itching, sneezing and nasal congestion. Its molecular formula is C_21_H_27_Cl_3_N_2_O_3_ and is rapidly absorbed with the gastro-intestinal track after the oral administration (15).

Literature reveal a variety of analytical methods for determination of CET alone or in combination with other drugs such as HPLC (16-20), TLC (21, 22), and spectrophotometry (23). There are numbers of investigation that compare the efficacy and safety of CET and MON which is used for treatment of pediatric perennial allergic rhinitis, seasonal allergic rhinitis and thyroid eye disease, alone or in combination (24-27). The results of previous studies demonstrate that CET was more effective than MON for rhinorrhea, sneezing and red eyes, whereas MON reveals a significant decrease in eosinophil levels in peripheral blood. However, combined MON/CET pretreatment significantly reduces the in-season symptom score for sneezing, eye itching, nasal itching, rhinorrhea and congestion.

Although combined MON/CET is more effective than each MON and CET alone in preventing eye itching rhinorrhea and nasal itching and delays the appearance of allergic rhinitis (AR) symptoms (25-27), to the best of our knowledge, there isn’t any commercial tablet for combined MON/CET. In addition, no reports were in literature on the separation and determination of MON and CET simultaneously by HPTLC method.

In this work, we describe the development of a sensitive and reliable HPTLC procedure for the simultaneous determination of MON and CET in authentic tablets. The results show that the proposed method is useful for the routine analysis of prepared formulations. With regards to the irregular prevalence of flu disease in the world, the need to produce more effective drugs seems to be essential. This research can help produce drugs with better performance.

## Experimental

*Materials and Reagent*

MON and CET standards (EP France) were received as a gift. Cetirizine and Montelukast tablets were purchased locally (Darou Pakhsh Mfg. Co.). Authentic tablets (labeled to contain MON 5 mg and CET 10 mg per tablet) were prepared in Darou Pakhsh research laboratory. All the chemicals and reagents used were of analytical grade and purchased from Merck or Fluka companies.

*Preparation of standard solutions*

Stock standard solutions of MON and CET were prepared by dissolving 20 mg of each substances in 100 mL methanol. The standard solution was prepared by dilution of 10 mL of each stock standard solution to 50 mL with methanol. The drugs were stable in methanolic solutions and no significant decreases in their concentration were observed after 12 h.

*Preparation of sample solutions*

Twenty tablets were weighed; their mean weight was calculated and finely powdered. A portion of powder equivalent to one tablet was weighed and dissolved in 100 mL methanol. The prepared sample solution was filtered with Whatman NO. 1 filter paper. Then, 10 mL of the solution was diluted to 50 mL. Final nominal concentration of MON and CET was 10 μg mL^-1^ and 20 μg mL^-1^ respectively and the solutions were stable up to 12 h.

*HPTLC conditions*

Chromatography was performed on silica gel HPTLC plates without prewashing. Aluminum HPTLC plates of 20 × 20 cm coated with 0.25 mm layers of silica gel 60 F_254 _(E. Merck, Germany) were used and developed in twin-trough glass chamber.

The samples were spotted in the form of 5 mm band widths by a Camag Linomat ΙV automatic TLC sampler at a distance of 20 mm from the edge of the plates. A constant application rate of 4 μL s^-1^ was employed and the space between two bands was 10 mm. The slit dimension was kept at 8 × 0.4 mm and 5 mm s^-1^ scanning speed was employed. The mobile phase consisted of ethyl acetate-methanol-ammonia solution (25%) (14:3:2 V/V/V) and the chamber saturation time was 20 min. Linear ascending development was carried out in the saturated twin-trough glass chamber up to a distance of 80 mm. After the development, the plates were air-dried at ambient temperature for 20 min. Densitometric scanning was performed in absorbance mode at 230 nm and the source of radiation utilized was deuterium lamp. Evaluation was done by using linear least-squares regression analysis method via peak areas. The HPTLC was carried out with a Camag TLC-Scanner ΙΙΙ (Camag Muttenz, Switzerland) fitted with a CATs software (version 4.04).

*Calibration curves of montelukast and cetirizine*

The calibration plots for MON and CET were constructed by analysis of ten solutions containing different concentrations of each drug. Each concentration was spotted five times on the HPTLC plates. Calibration plots of peak area against respective amount were established separately for MON and CET using linear least square regression.

## Results and Discussion

Experimental conditions such as mobile phase and wavelength of detection were optimized. The selection of wavelength was based on UV absorbance of both drugs.

**Figure 2:**
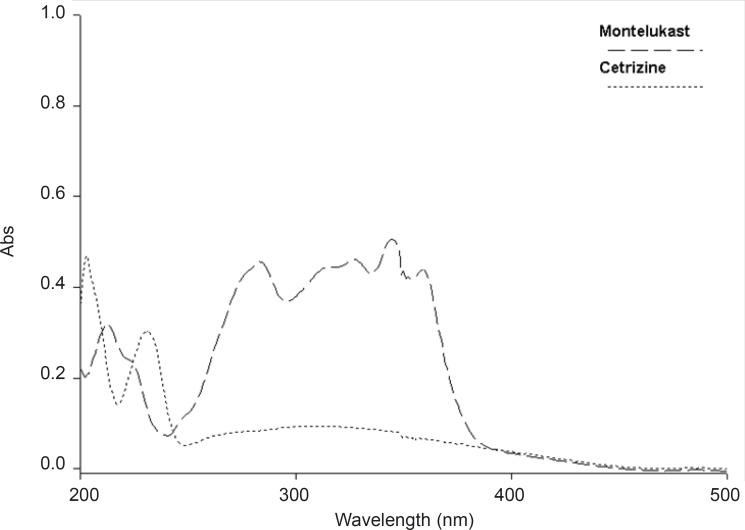
UV absorption spectra of CET (4 μg mL^-1^) and MON (10 μg mL^-1^) in methanol

The wavelength of scanning was chosen to be 230 nm (Figure 2). 

**Figure 3:**
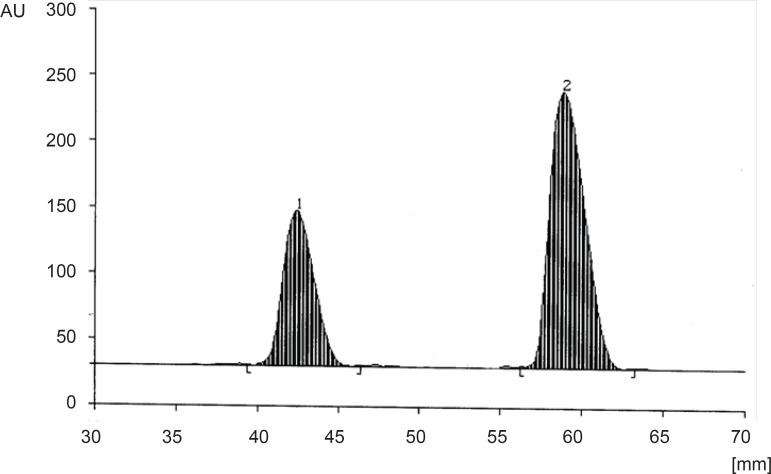
Densitogram of CET (1520 ng spot^-1^); peak 1 (R_f _= 0.30 ± 0.01) and MON (1520 ng spot^-1^); peak 2 (R_f_ = 0.52 ± 0.02) in standard solutions.

Different mobile phases containing ethyl acetate, methanol, and ammonia solution (25%) in different proportions were examined. The greatest differences between the R_f_-values of CET (R_f _= 0.3) and MON (R_f_ = 0.52) with minimal tailing were obtained by using the mobile phase consisting of ethyl acetate : methanol : ammonia solution (25%) (14 : 3 : 2 v/v/v) (Figure 3).

*Linearity*

The linearity of plotting peak area of different concentrations for MON and CET was investigated over the concentration range of 40-4000 ng spot^-1^. Results indicate that calibration plots were found to be linear over the range of 120-1000 ng spot^-1^ for MON (6 points) and 40-2000 ng spot^-1^ for CET (8 points). Furthermore, the analysis of heights of chromatogram peaks also showed linear relationship for MON and CET (Table 1). The plot obtained from residuals analysis (not mentioned here) did not show a visible trend and was randomly scattered.

**Table 1:** Characteristic parameters for linear regression equations of MON and CET of the method.

**Parameter**	**MON**		**CET**	
	**Area**	**height**	**Area**	**Height**
Linearity range (μg spot^-1^)	120-1000	120-1000	40-2000	40-2000
Regression equations (Y)*	Y=75.67 + 3.600X	Y=29.50 + 0.097X	Y=211.90 + 1.902X	Y=10.40 + 0.085X
First regression coefficient (b)	3.600	0.0978	1.902	0.0854
Standard deviation of the slope (S_b_)%	0.390	0.011	0. 179	0.030
Intercept (a)	75.67	29.50	211.90	10.40
Standard deviation of the Intercept (S_a_)%	8.16	9.66	15.88	2.90
Correlation coefficient	R^2^ = 0.9968	R^2^ = 0.9917	R^2^ = 0.9961	R^2^ = 0.9943

* *Y= a + bX*, whereis *X *the concentration of the drug in μg spot^-1^ and *Y *is the peak area of the spot

*Precision*

The precision of the proposed method was verified by measuring of repeatability or intra-day variation and intermediate precision or inter-day variation. To study intra-day variation, six replicates of sample solutions containing MON (500 ng spot^-1^) and CET (1000 ng spot^-1^) were analyzed on the same day. To study inter-day variation, analysis of three replicates of sample solutions with the same concentration was performed on three different days. Intra-day variation as RSD was 1.09% for MON and 1.51% for CET, and inter-day variation as RSD was 1.31% for MON and 1.63% for CET. The low values of RSD show that the method is precise.

*Accuracy (recovery)*

The accuracy of the method was obtained on the basis of recovery studies performed by standard addition at 80, 100 and 120% of the label claim each in triplicate. A known amount of each standard powder was mixed with sample of tablet powder and these were then analyzed as described above. The results from recovery analysis are given in Table 2. As could be seen, the obtained recoveries were ranged from 99.20 to 100.88% with RSD ranging from 1.02 to 1.90% for MON and 98.13 to 100.05% with RSD ranging from 1.57 to 1.85% for CET.

**Table 2:** Results from recovery analysis for MON and CET (n = 5).

**Level (%)**	**Theoretical content (mg)**		**Amount added (mg)**		**Mean amount found (mg)**		**Recovery (%)**		**RSD (%)**	
	**MON**	**CET**	**MON**	**CET**	**MON**	**CET**	**MON**	**CET**	**MON**	**CET**
0	5	10	0	0	4.96	9.87	99.20	99.70	1.02	1.68
80	5	10	4	8	9.08	18.01	100.88	100.05	1.90	1.85
100	5	10	5	10	10.03	19.82	100.30	99.10	1.83	1.57
120	5	10	6	12	10.98	21.59	99.81	98.13	1.73	1.78

Theoretical content (mg).

*Limit of detection and limit of quantification*

In order to estimate the limit of detection (LOD) and limit of quantification (LOQ), blank methanol was spotted five times following the same method as explained before and the standard deviation (S_b_) of the magnitude of analytical response was determined. The LOD was expressed as 3S_b_/slope of the calibration curve and the LOQ was expressed as 10S_b_/slope (28, 29). The LODs were found to be 2.08 and 3.94 ng spot^-1^, for MON and CET respectively. The LOQs were found to be 6.94 and 13.14 ng spot^-1^ for MON and CET respectively, which indicate the adequate sensitivity of the method.

*Specificity*

The specificity of the method was determined by analysis of standard and test samples of drugs. The spots for both drugs were confirmed by comparing the R_F_-values and spectra of the sample spots with those of standard drugs. Good correlation between the corresponding spectra indicated that the method is specific for these drugs and no other tablet component interferes with these drugs.

*Robustness*

The robustness of the method was studied by applying small variations in chamber saturation time (± 10%), mobile phase composition (± 2%), time after spotting and before development (0, 15, 30 and 60 min) and time after development and before scanning (0, 15, 30 and 60 min). The robustness of the method was determined at concentrations of 500 ng spot^-1^ and 1000 ng spot^-1^ for MON and CET respectively and the effects of variation on the peak area of the drugs were studied. The standard deviation of peak areas of the drugs was studied and found to be < 2% which confirms the robustness of the proposed method (Table 3).

**Table 3:** Results from testing of robustness as RSD (%) of peak area

**Conditions varied**	**MON**	**CET**
Chamber saturation time (min)	0.69	0.96
Mobile phase composition (%)	1.93	1.31
Time after spotting and before development (min)	1.24	1.02
Time after development and before scanning (min)	1.76	1.53

*Analysis of tablet formulation*

Ten μL of sample stock solution was applied to an HPTLC plate to give final amount of 100 ng spot^-1 ^for MON and 200 ng spot^-1 ^for CET. After the chromatographic development, peak areas of the bands were measured and amount of each drug was estimated from the respective calibration plots. The procedure was repeated six times. The average assays (n = 6) were 99.51 ± 0.39 and 100.19 ± 0.67 for MON and CET respectively.

## Conclusion

In general, the HPTLC method has some advantages over HPLC, which commonly used for analysis of pharmaceuticals, such as large sample capacity, short run time, and minimal volume use of solvents. As mentioned before, combined MON/CET treatment can be significantly effective for rhinorrhea and sneezing and determinations with such a simple HPTLC method can provide new ways for quality control analysis of more effective industrial productions. The proposed HPTLC method provides simple, accurate and reproducible quantitative analysis for simultaneous determination of MON and CET in bulk drugs and pharmaceutical formulations without interference from excipients. Both peak area and peak height can be used for analysis and it may be extended to study the degradation kinetics of drugs. With applying the proposed method, one can gain the advantages of speed, lower cost and environmental protection without sacrificing the accuracy.
